# Asymptomatic Multiple Bilateral Segmental Pulmonary Embolisms Caused by Cement After Multilevel Vertebroplasty: A Case Report and Literature Review

**DOI:** 10.1155/cris/7151548

**Published:** 2026-08-31

**Authors:** Elie Mokled, Anthony Sadek, Maher Ghandour, Fatima Farhat, Amer Abdallah

**Affiliations:** ^1^ Faculty of Medical Science, Lebanese University, Beirut, Lebanon, ul.edu.lb; ^2^ Department of Orthopedic Surgery, Hopital Libanais Geitaoui CHU, Beirut, Lebanon, hopital-libanais.com; ^3^ Orthopedics Department, Aachen University Hospital, Aachen, Germany, ukaachen.de; ^4^ Department of Diagnostic Imaging and Interventional Therapeutics, Hopital Libanais Geitaoui CHU, Beirut, Lebanon, hopital-libanais.com

**Keywords:** bone cements, polymethyl methacrylate, pulmonary embolism, spinal fractures, vertebroplasty

## Abstract

Pulmonary cement embolization (PCE) is a recognized, often asymptomatic complication of percutaneous vertebroplasty (PVP), a procedure commonly used to stabilize vertebral compression fractures. Here, we report the case of a 58‐year‐old male who, after vertebroplasty for multiple acute thoracic and lumbar fractures, exhibited segmental, peripheral PCE on postoperative imaging. Despite cement leakage into the pulmonary vasculature, the patient remained asymptomatic, stable, and was discharged following brief observation. A review of the literature reveals varied management approaches for asymptomatic PCE, from conservative monitoring to anticoagulation therapy, with outcomes generally favorable regardless of intervention. Our case supports the safety of conservative management in asymptomatic PCE, highlighting the need for standardized guidelines to aid clinical decision‐making. Early detection through postoperative imaging, combined with tailored patient management, may improve outcomes while minimizing unnecessary interventions for asymptomatic PCE. Emergency physicians should be aware of PCE as a potential postoperative complication of vertebroplasty, even in asymptomatic patients, to ensure timely recognition, appropriate monitoring, and avoid unnecessary interventions.

## 1. Introduction

Vertebroplasty is a minimally invasive procedure commonly used for stabilizing vertebral compression fractures, often resulting from osteoporosis, trauma, or malignancy. Despite its efficacy, complications such as pulmonary cement embolism (PCE) can occur due to leakage of polymethyl methacrylate (PMMA) into the venous system [1, 2]. PCE has been reported to occur in 11%–76% of patients undergoing vertebroplasty and in 4.8%–39% of those undergoing kyphoplasty [3]. Although PCE is frequently asymptomatic (reported in 9%–25.7% of cases) [4–6], it can lead to significant morbidity and, in rare cases, mortality [7, 8]. This case report discusses an asymptomatic PCE episode following vertebroplasty and provides a literature review to contextualize this occurrence.

## 2. Case Report

A 58‐year‐old male presented to the emergency department following electrocution and a subsequent fall from standing height. His medical history included epilepsy managed with Tegretol and a history of smoking. The patient had no surgical history or known allergies. Upon arrival, the patient reported diffuse lower back pain and impaired mobility. His vital signs were stable, and physical examination revealed tenderness over the thoracic and upper lumbar spine without neurological deficits.

Initial investigations included laboratory tests, a computed tomography (CT) scan of the brain, and full‐spine X‐rays. The laboratory results were unremarkable. The CT brain scan showed no acute changes, while spinal imaging revealed multiple vertebral fractures. Specifically, there was a severe T5 compression fracture with a 60% height loss, T11 compression with a 50% height loss, and minor compressions at T12 (15%) and L1 (35%). Chest imaging revealed clear lung fields without pleural effusion.

**Figure 1:**
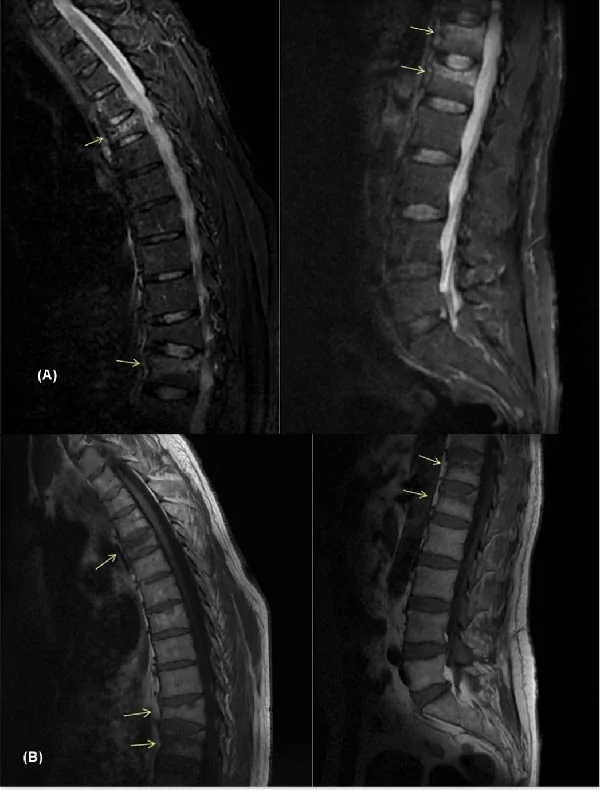
Sagittal MRI of the thoracolumbar spine showing multiple vertebral compression fractures. (A) Short tau inversion recovery (STIR) sequence demonstrating acute fractures at T5, T12, and L1 (yellow arrows) with marrow edema indicating acuity, and a subacute T11 fracture showing intermediate signal. (B) Corresponding T1‐weighted images showing hypointense signal at T5, T11, T12, and L1, consistent with acute and subacute fractures.

**Figure 2:**
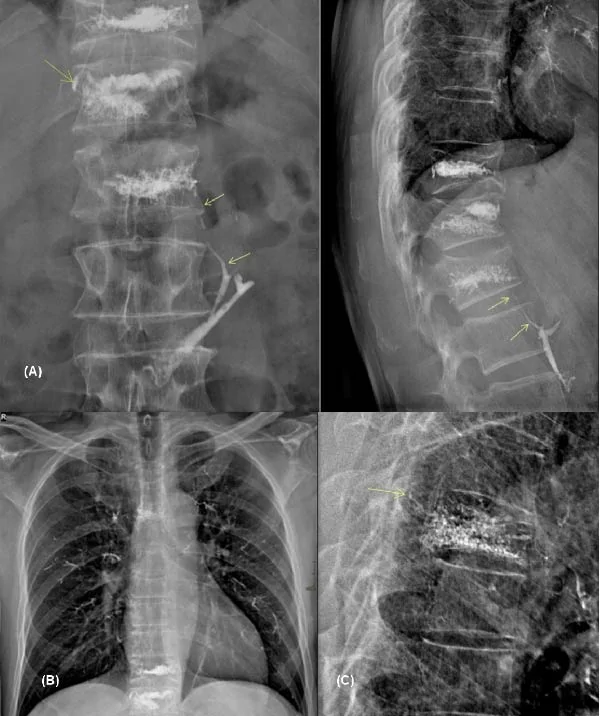
Radiographic images of the thoracolumbar spine following vertebral augmentation. (A) Anteroposterior and sagittal lumbar radiographs demonstrating vertebroplasty/kyphoplasty cement opacification at T11, T12, and L1 (yellow arrows) with no posterior wall retropulsion. (B) Frontal chest radiograph showing cemented T5 vertebral level (yellow arrow). (C) Magnified lateral thoracic view confirming intact posterior vertebral wall and stable cement distribution at T5.

Magnetic resonance imaging (MRI) of the thoracolumbar spine confirmed acute fractures at T5, T12, and L1, with a subacute fracture at T11, without posterior wall retropulsion (Figures 1 and 2). Vertebroplasty was planned to stabilize the spine and manage the pain. Using a monopedicular approach, 4 cc of PMMA was injected into each fractured vertebra. An expert surgeon performed all procedures, and the cement was not in fluid form or injected under pressure. The procedure was completed without any immediate complications, and the patient regained motor function in both lower limbs.

**Figure 3:**
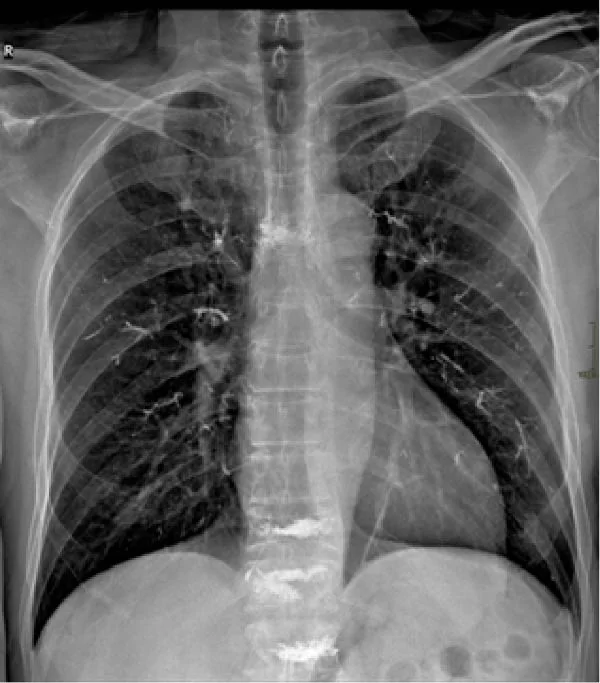
Anteroposterior X‐ray view of the thoracic and lumbar spine, showing vertebroplasty at T5, T11, T12, and L1 levels with the presence of multiple bilateral segmental peripheral pulmonary emboli caused by polymethylmethacrylate (PMMA).

**Figure 4:**
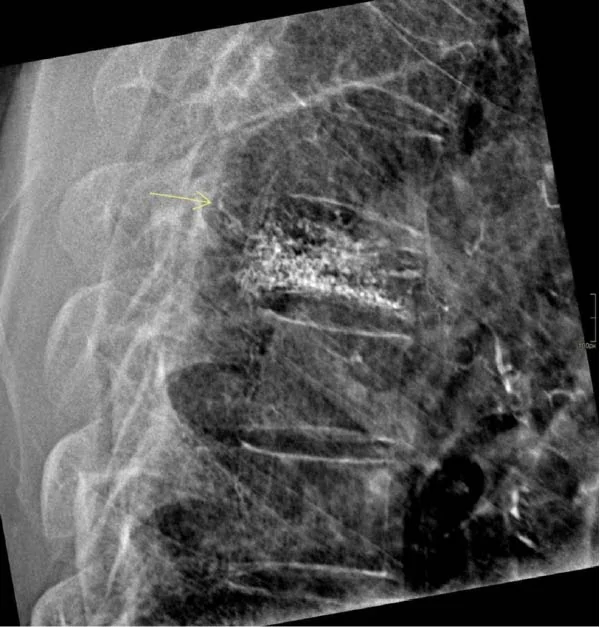
Lateral X‐ray view of the thoracic spine showing T5 polymethylmethacrylate (PMMA) extravasation (yellow arrow).

**Figure 5:**
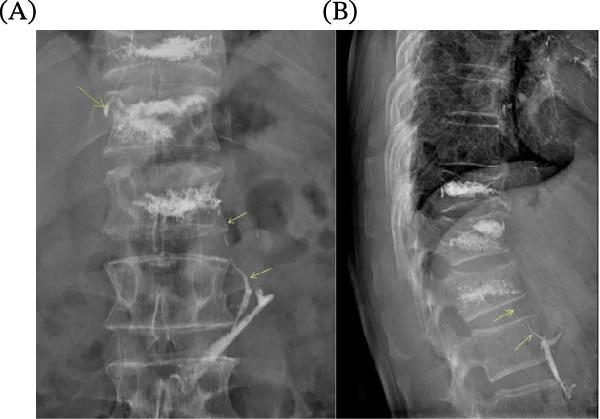
Anteroposterior (A) and lateral (B) X‐ray thoracolumbar spine X‐ray views showing T12 and L1 polymethylmethacrylate (PMMA) extravasation (yellow arrow).

Postoperative thoracic and lumbar spine X‐rays performed 4 h later demonstrated successful vertebroplasty at T5, T11, T12, and L1. However, cement leakage into the paravertebral veins was noted, resulting in multiple bilateral segmental peripheral PCE (Figures 3–5). The diagnosis of PCE was established on plain radiographs, on which the emboli appeared as characteristic tubular and branching radiopacities of cement, following the course of the pulmonary vasculature (47). Given the patient’s asymptomatic and hemodynamically stable presentation, CT pulmonary angiography was not performed, and there was no clinical or radiographic evidence of concomitant thrombotic (venous) pulmonary embolism. Despite this, the patient remained hemodynamically stable, asymptomatic, and was discharged on pain medication after 24 h of observation, with follow‐up scheduled for 1 week later. At that 1‐week follow‐up, the patient remained clinically well and asymptomatic; the assessment was clinical, and no control (follow‐up) imaging was obtained.

## 3. Discussion

PCE is a known complication of percutaneous vertebroplasty (PVP), albeit often asymptomatic [4, 5]. The present case illustrates this asymptomatic pattern: despite radiographically evident cement leakage into the paravertebral veins, the patient remained hemodynamically stable and required only routine monitoring before discharge.

Our findings align with the broader literature, which reports asymptomatic presentations in many cases of PCE [9, 10]. The literature review (Table 1) reveals that asymptomatic PCE cases often proceed with uneventful recoveries, regardless of therapeutic intervention, which varies widely across cases [4, 5, 11–47]. For instance, studies have documented successful outcomes without anticoagulation therapy [48], while other cases opted for anticoagulation as a preventive measure against thromboembolic complications [40]. The management of asymptomatic PCE is thus not standardized and appears influenced by factors such as patient comorbidities, extent of embolization, and physician preference [49].

**Table 1:** Literature review of asymptomatic cases presenting with pulmonary cement embolization after vertebroplasty.

Author	YOP	Number of patients	Number of asymptomatic PCE	Age	Gender	Procedure/indication	Time from PVP to PCE	PE Type	Number of PCE	Biggest PCE size (mm)	Therapy	Outcome
Amar [11]	2001	3	2	—	—	PVP/unclear	—	—	—	—	—	Uneventful recovery
Jang [12]	2002	3	1	60	F	PVP/myeloma	—	—	—	—	LDH	Uneventful recovery
Bernhard [13]	2003	1	1	—	—	PVP/osteoporotic fracture	—	—	—	—	—	—
Pleser [14]	2004	1	1	—	—	PVP/osteoporotic fracture	—	—	—	—	Heparin + Coumarin for 6 mo	—
		1	1	80	F	PVP/osteoporotic fracture	—	—	—	—	Anticoagulation	Discharged
Seo [15]	2005	1	1	—	—	PVP/osteoporotic fracture	—	—	—	—	Embolectomy	—
Mac Taggert [16]	2006	1	1	—	—	PVP/osteoporotic fracture	—	—	—	—	—	—
		1	1	65	F	PVP/multiple myeloma	—	—	—	—	None	Discharged
Neuwirth [17]	2006	1	1	—	—	PVP/osteoporotic fracture	—	—	—	—	—	—
Baumann [18]	2006	1	1	50	F	PVP/osteoporotic fracture	—	—	—	—	Coumarin for 3 mo	Discharged
Freitag [19]	2006	1	1	—	—	PVP/osteoporotic fracture	—	—	—	—	Coumarin for 6 mo	—
Quesada [20]	2006	1	1	—	—	PVP/osteoporotic fracture	—	—	—	—	—	—
Bonardel [21]	2007	1	1	61	M	PVP/osteoporotic fracture	—	—	—	—	Coumarin for 6 mo	Uneventful recovery
Abdul‐Jalil [22]	2007	2	1	64	F	PVP/osteoporotic fracture	—	—	—	—	LDH	Discharged
Schneider [23]	2007	1	1	—	—	PVP/osteoporotic fracture	—	—	—	—	—	—
Cadeddu [24]	2009	1	1	68	F	PVP/osteoporotic fracture	—	—	—	55x4	Right cardiac catheterization; failed cement removal	Discharged
Akinola [25]	2010	1	1	76	M	PVP/severe scoliosis	—	—	—	—	Anticoagulation	Uneventful recovery
Venman [5]	2010	202	52	—	—	PVP	—	—	—	—	—	—
Leutmer [4]	2011	244	22	64.6 (11.6)	M/*F* = 7/15	PVP/‐	—	—	—	—	—	—
Matouk [26]	2012	1	1	83	F	PVP/osteoporotic fracture	—	—	—	—	None	Discharged
Mishriki [27]	2012	1	1	50	F	PVP/osteoporotic fracture	—	—	—	—	—	—
Garcia‐Fontan [28]	2013	1	1	69	M	PVP/compression fracture	—	—	—	—	—	—
Geraci [29]	2013	1	1	70	M	PVP/osteoporotic fracture	—	—	—	—	Anticoagulant + Antibiotic + short‐term corticosteroids	Uneventful recovery
Giraldo [30]	2013	1	1	74	M	PVP/osteoporotic fracture	—	—	—	—	None	Discharged
Chen [31]	2014	1	1	39	F	PVP/fracture due to bone metastasis	—	—	—	—	None	Discharged
Zhao [32]	2014	1	1	55	F	PVP/osteoporotic fracture	—	—	—	—	Percutaneous retrieval of cement	Uneventful recovery
Guirguis [33]	2015	1	1	72	M	PVP/painful fracture	—	—	—	—	None	Discharged
Nooh [34]	2015	1	1	70	F	PVP/compression fracture	9 months	—	—	—	Interventional removal	Discharged
Chai [35]	2016	1	1	51	F	PVP/fracture due to bone metastasis	—	—	—	—	Anticoagulant	Discharged
Gabe [36]	2016	1	1	58	F	PVP/osteoporotic fracture	—	—	—	—	None	Discharged
Gorospe [37]	2016	1	1	58	F	PVP/fracture due to multiple myeloma	—	—	—	—	None	Discharged
Memarpour [38]	2016	1	1	32	M	PVP/‐	—	—	—	—	None	Discharged
Chang [39]	2017	1	1	59	M	PVP/traumatic compression fracture	—	—	—	—	Anticoagulant	Discharged
Mansour [40]	2018	102	1	78	F	PVP/osteoporotic fracture	—	Segmental	—	—	None	Alive
			1	55	M	PVP/osteoporotic fracture	—	Lobar	—	—	None	Dead (12 mo)
			1	81	F	PVP/osteoporotic fracture	—	Subsegmental	—	—	LMWH	Alive
			1	37	M	PVP/malignancy	—	Subsegmental	—	—	None	Dead
			1	50	F	PVP/malignancy	—	Subsegmental	—	—	None	Dead (24 mo)
			1	63	M	PVP/malignancy	—	Lobar	—	—	None	Dead (20 mo)
			1	50	M	PVP/malignancy	—	Subsegmental	—	—	None	Alive
			1	60	F	PVP/malignancy	—	Subsegmental	—	—	LMWH	Dead (11 mo)
Kong [41]	2019	12	1	70	F	PVP/‐	—	—	3	50x2	Percutaneous retrieval of cement	Discharged (day 7)
			1	77	F	PVP/‐	—	—	1	70x2	Percutaneous retrieval of cement	Discharged (day 5)
			1	88	F	PVP/‐	—	—	7	16x4	Observation	Discharged (day 3)
			1	61	F	PVP/‐	—	—	1	50x3	Observation	Discharged (day 4)
			1	84	F	PVP/‐	—	—	1	23x1	Observation	Discharged (day 4)
			1	79	F	PVP/‐	—	—	8	7x2	Observation	Discharged (day 5)
			1	70	M	PVP/‐	—	—	5	20x2	Observation	Discharged (day 3)
			1	80	F	PVP/‐	—	—	5	4x2	Observation	Discharged (day 4)
			1	84	F	PVP/‐	—	—	1	8x2	Observation	Discharged (day 5)
			1	79	F	PVP/‐	—	—	1	6x3	Observation	Discharged (day 4)
			1	68	F	PVP/‐	—	—	1	33x2	Observation	Discharged (day 3)
			1	82	F	PVP/‐	—	—	2	16x2	Observation	Discharged (day 5)
Ross [42]	2021	1	1	46	F	PVP/benign lytic lesion		Segmental	—	50x1	Subcutaneous LMWH + DOACs	Under annual follow‐up
Badillo [43]	2022	1	1	56	F	PVP/bone metastasis		—	—	—	None	—
Lenga [44]	2024	104	27	—	—	—	—	—	—	—	—	—
Fish [45]	2023	1	1	50	M	PVP/fracture due to multiple myeloma	4 weeks	Segmental	—	—	Anticoagulation	Discharged
Beup [46]	2023	1	1	76	F	PVP/bone metastasis		—	—	—	Enoaparin >> Apixaban	Discharged
Ateş [47]	2021	1	1	73	M	PVP/compression fracture		Segmental	—	—	Anticoagulant	—

*Note*: — indicates that the data were not sufficiently reported.

Abbreviations: DOAC, direct oral anticoagulants; F, female; LDH, low‐dose heparin; LMWH, low‐molecular weight heparin; M, male; mo, month; PCE, pulmonary cement embolization; PVP, percutaneous vertebroplasty; YOP, year of publication.

Interestingly, our case adds to a body of evidence that suggests that patient stability post‐PVP may justify conservative management for asymptomatic PCE cases. Observational strategies, as seen in some of the reviewed literature, might suffice for small, peripheral emboli, especially when patients exhibit no hemodynamic or respiratory compromise [40]. The risks associated with prolonged anticoagulation, such as bleeding complications, should be weighed carefully against potential benefits, especially in patients without symptoms. Furthermore, some reports advocate for short‐term anticoagulation, but evidence regarding its necessity and efficacy remains inconclusive [50].

In our case, the involvement of multilevel thoracolumbar fractures, including T5, might be an aggravating factor for PCE, as suggested in the literature [51]. The thoracic vertebrae, particularly around T5, are theorized to be more susceptible due to their anatomical proximity to critical venous structures that facilitate cement migration into the pulmonary vasculature. The pathophysiology of PCE involves the entry of PMMA into the vertebral venous system through the basivertebral veins or fracture clefts before the cement is fully cured. Although this is frequently attributed to high‐pressure injection of low‐viscosity cement, extravasation can also occur in the absence of forceful, fluid‐phase injection. In the present case, an experienced operator injected cement that was neither in a fluid state nor delivered under pressure, yet embolism still occurred. This is consistent with the concept that the cumulative rise in intravertebral pressure generated by the sequential augmentation of multiple levels (T5, T11, T12, and L1), even with ideal cement viscosity and in expert hands, can be sufficient to drive cement into the paravertebral venous plexus [6]. This risk is further heightened when the cortical bone is fractured. Moreover, some anatomical studies have suggested potential communication between thoracic vertebral venous networks and pulmonary arteries [52], which may further exacerbate the risk of cement migration to the lungs. Our patient’s complex fracture patterns with significant vertebral body compression might have predisposed him to PCE. This aligns with the literature that the risk of PCE is heightened in thoracic over lumbar levels [53], as their proximity to the paravertebral veins provides easy access to the bloodstream. Multilevel augmentation (three or more levels) and a unipedicular puncture approach, both present in our patient, have likewise been reported as independent risk factors for PCE [6]. Additionally, the amount of PMMA seems to contribute to the risk, with higher volumes showing a higher likelihood of PCE (especially above 3.5 cc) [54]. Identifying patients at elevated risk and refining cement injection techniques could mitigate this risk.

## 4. Why Should an Emergency Physician Be Aware of This?

Emergency physicians play a critical role in the initial recognition and triage of patients following vertebroplasty, and awareness of asymptomatic PCE as a possible postoperative finding is essential to guide observation decisions, avoid overtreatment, and contribute to a multidisciplinary management approach.

In conclusion, our case supports the notion that asymptomatic PCE after PVP can be managed conservatively in select patients, particularly those who are hemodynamically stable. Routine postoperative imaging may be warranted to detect PCE in high‐risk patients. Future studies should aim to establish clear guidelines for asymptomatic PCE management, helping clinicians balance intervention risks with potential benefits.

## Funding

This research did not receive any specific grant from funding agencies in the public, commercial, or not‐for‐profit sectors.

## Consent

Written informed consent was obtained from the patient for the publication of anonymized clinical details included in the manuscript.

## Conflicts of Interest

The authors declare no conflicts of interest.

## Data Availability

The data used to support the findings of this study are available from the corresponding author upon request.

## References

[1] Li S.-F. , Li X.-Y. , and Bai X.-H. , et al.A Meta-Analysis Comparing the Efficacy of Mineralized Collagen-Polymethylmethacrylate and Polymethylmethacrylate Bone Cements in the Treatment of Vertebral Compression Fractures, PLoS ONE. (2024) 19, no. 3, 10.1371/journal.pone.0299325. PMC10923492 38457423

[2] McGarvey C. R. , Nair A. , Nawras Y. , Oenick J. , and Vattipally V. R. , Cement Embolism After Kyphoplasty, Cureus. (2024) 16, no. 1, 10.7759/cureus.52821. PMC10883896 38406080

[3] Armsen N. and Boszczyk B. , Vertebro-/Kyphoplasty History, Development, Results, European Journal of Trauma. (2005) 31, no. 5, 433–441, 10.1007/s00068-005-2103-z.

[4] Luetmer M. T. , Bartholmai B. J. , Rad A. E. , and Kallmes D. F. , Asymptomatic and Unrecognized Cement Pulmonary Embolism Commonly Occurs With Vertebroplasty, American Journal of Neuroradiology. (2011) 32, no. 4, 654–657, 10.3174/ajnr.A2368. 21415145 PMC7965890

[5] Venmans A. , Klazen C. A. H. , and Lohle P. N. M. , et al.Percutaneous Vertebroplasty and Pulmonary Cement Embolism: Results From VERTOS II, American Journal of Neuroradiology. (2010) 31, no. 8, 1451–1453, 10.3174/ajnr.A2127. 20488908 PMC7966118

[6] Wang L. , Lu M. , and Zhang X. , et al.Risk Factors for Pulmonary Cement Embolism After Percutaneous Vertebroplasty and Radiofrequency Ablation for Spinal Metastases, Frontiers in Oncology. (2023) 13, 10.3389/fonc.2023.1129658, 1129658. 37213292 PMC10196379

[7] Opriță B. , Ghinea G.-L. , Dinu A.-B. , and Opriță R. , Hemodynamic Instability From Cement Pulmonary Embolism Following Vertebroplasty: A Case Report, Reports. (2025) 8, no. 3, 10.3390/reports8030172, 172. 40981130 PMC12452359

[8] Pu J. , He J.-Q. , Yi Q. , and Zhou H. , Simultaneous Pulmonary and Intracardiac Bone Cement Embolism After Vertebroplasty: A Case Report and Literature Review, Journal of Cardiothoracic Surgery. (2025) 20, no. 1, 10.1186/s13019-025-03654-w, 405. 41153029 PMC12570630

[9] Rose L. D. , Bateman G. , and Ahmed A. , Clinical Significance of Cement Leakage in Kyphoplasty and Vertebroplasty: A Systematic Review, European Spine Journal. (2024) 33, no. 4, 1484–1489, 10.1007/s00586-023-08026-3. 37999769

[10] Tjønnfjord E. , Ho A. P. T. , Sætre D. O. , and Raouf N. , Pulmonary Cement Embolism: A Rare Complication of Vertebroplasty, Journal of International Medical Research. (2024) 52, no. 11, 10.1177/03000605241291447, 3000605241291447. 39501724 PMC11539260

[11] Amar A. P. , Larsen D. W. , Esnaashari N. , Albuquerque F. C. , Lavine S. D. , and Teitelbaum G. P. , Percutaneous Transpedicular Polymethylmethacrylate Vertebroplasty for the Treatment of Spinal Compression Fractures, Neurosurgery. (2001) 49, no. 5, 1105–1115. 11846904

[12] Jang J. S. , Lee S. H. , and Jung S. K. , Pulmonary Embolism of Polymethylmethacrylate After Percutaneous Vertebroplasty: A Report of Three Cases, Spine. (2002) 27, no. 19, E416–E418, 10.1097/00007632-200210010-00021. 12394937

[13] Bernhard J. , Heini P. F. , and Villiger P. M. , Asymptomatic Diffuse Pulmonary Embolism Caused by Acrylic Cement: An Unusual Complication of Percutaneous Vertebroplasty, Annals of the Rheumatic Diseases. (2003) 62, no. 1, 85–86, 10.1136/ard.62.1.85. 12480681 PMC1754288

[14] Pleser M. , Roth R. , Wörsdörfer O. , and Manke C. , et al.Pulmonary Embolism Caused by PMMA in Percutaneous Vertebroplasty: Case Report and Review of the Literature, Der Unfallchirurg. (2004) 107, 807–811. 15083278 10.1007/s00113-004-0763-5

[15] Seo J. S. , Kim Y. J. , Choi B. W. , Kim T. H. , and Choe K. O. , MDCT of Pulmonary Embolism After Percutaneous Vertebroplasty, American Journal of Roentgenology. (2005) 184, no. 4, 1364–1365, 10.2214/ajr.184.4.01841364. 15788629

[16] MacTaggart J. N. , Pipinos I. I. , Johanning J. M. , and Lynch T. G. , Acrylic Cement Pulmonary Embolus Masquerading as an Embolized Central Venous Catheter Fragment, Journal of Vascular Surgery. (2006) 43, no. 1, 180–183, 10.1016/j.jvs.2005.09.002. 16414409

[17] Neuwirth J. , Weber J. , and Kohler B. , Pulmonary Cement Embolism After Vertebroplasty in Multiple Osteoporotic Vertebral Fractures, DMW - Deutsche Medizinische Wochenschrift. (2006) 131, no. 41, 2275–2278, 10.1055/s-2006-951363.

[18] Baumann A. , Tauss J. , Baumann G. , Tomka M. , Hessinger M. , and Tiesenhausen K. , Cement Embolization Into the Vena Cava and Pulmonal Arteries After Vertebroplasty: Interdisciplinary Management, European Journal of Vascular and Endovascular Surgery. (2006) 31, no. 5, 558–561, 10.1016/j.ejvs.2005.11.008. 16376118

[19] Freitag M. , Gottschalk A. , Schuster M. , Wenk W. , Wiesner L. , and Standl T. G. , Pulmonary Embolism Caused by Polymethylmethacrylate During Percutaneous Vertebroplasty in Orthopaedic Surgery, Acta Anaesthesiologica Scandinavica. (2006) 50, no. 2, 248–251, 10.1111/j.1399-6576.2005.00821.x. 16430551

[20] Quesada N. and Mutlu GkM. , Pulmonary Embolization of Acrylic Cement During Vertebroplasty, Circulation. (2006) 113, no. 8, e295–e296, 10.1161/CIRCULATIONAHA.105.557017. 16505181

[21] Bonardel G. , Pouit B. , and Gontier E. , et al.Pulmonary Cement Embolism After Percutaneous Vertebroplasty, Clinical Nuclear Medicine. (2007) 32, no. 8, 603–606, 10.1097/RLU.0b013e3180a1ad5a. 17667431

[22] Abdul-Jalil Y. , Bartels J. , Alberti O. , and Becker R. , Delayed Presentation of Pulmonary Polymethylmethacrylate Emboli After Percutaneous Vertebroplasty, Spine. (2007) 32, no. 20, E589–E593, 10.1097/BRS.0b013e31814b84ba. 17873801

[23] Schneider L. and Plit M. , Pulmonary Embolization of Acrylic Cement During Percutaneous Vertebroplasty, Internal Medicine Journal. (2007) 37, no. 6, 423–425, 10.1111/j.1445-5994.2007.01377.x. 17535394

[24] Cadeddu C. , Nocco S. , Secci E. , Deidda M. , Pirisi R. , and Mercuro G. , Echocardiographic Accidental Finding of Asymptomatic Cardiac and Pulmonary Embolism Caused by Cement Leakage After Percutaneous Vertebroplasty, European Journal of Echocardiography. (2009) 10, no. 4, 590–592, 10.1093/ejechocard/jep030. 19329500

[25] Akinola B. , Lutchman L. , Barker P. , and Rai A. , Pulmonary Cement Embolism During Cement Augmentation of Pedicle Screw Fixation: A Case Report, Journal of Orthopaedic Surgery. (2010) 18, no. 3, 364–366, 10.1177/230949901001800322. 21187553

[26] Matouk C. C. , Krings T. , Ter Brugge K. G. , and Smith R. , Cement Embolization of a Segmental Artery After Percutaneous Vertebroplasty: A Potentially Catastrophic Vascular Complication, Interventional Neuroradiology. (2012) 18, no. 3, 358–362, 10.1177/159101991201800318. 22958778 PMC3442313

[27] Mishriki Y. Y. and Hallinan B. , Puzzles in Practice, Postgraduate Medicine. (2015) 124, no. 1, 174–176, 10.3810/pgm.2012.01.2530. 22416294

[28] García-Fontán E. , Blanco Ramos M. , and Obeso Carillo G. A. , Cement Embolism During a Kyphoplasty, European Journal of Cardio-Thoracic Surgery. (2013) 44, no. 1, 10.1093/ejcts/ezs715. 23337329

[29] Geraci G. , Lo Iacono G. , Lo Nigro C. , Cannizzaro F. , Cajozzo M. , and Modica G. , Asymptomatic Bone Cement Pulmonary Embolism After Vertebroplasty: Case Report and Literature Review, Case Reports in Surgery. (2013) 2013, no. 1, 10.1155/2013/591432, 591432. 23738182 PMC3662203

[30] Sifuentes Giraldo W. A. , Lamúa Riazuelo J. R. , Gallego Rivera J. I. , and Vázquez Díaz M. , Cement Pulmonary Embolism After Vertebroplasty, Reumatología Clínica. (2013) 9, no. 4, 239–242, 10.1016/j.reumae.2013.03.003. 23481509

[31] Chen C.-Y. , Cheng W.-E. , Lin C.-H. , Chen S.-C. , and Shih C.-M. , Tadpoles in the Lungs. Cement Pulmonary Embolism Complicating Vertebroplasty of Spinal Metastasis, American Journal of Respiratory and Critical Care Medicine. (2014) 190, no. 3, 340–341, 10.1164/rccm.201403-0433IM. 25084261

[32] Zhao Y. , Liu T. , Zheng Y. , Wang L. , and Hao D. , Successful Percutaneous Retrieval of a Large Pulmonary Cement Embolus Caused by Cement Leakage During Percutaneous Vertebroplasty: Case Report and Literature Review, Spine. (2014) 39, no. 26, E1616–E1621, 10.1097/BRS.0000000000000613. 25271513

[33] Guirguis M. S. and Shroff G. S. , Cement Pulmonary Embolism, The American Journal of the Medical Sciences. (2015) 349, no. 5, 10.1097/MAJ.0000000000000211. 24534786

[34] Nooh A. , Abduljabbar F. H. , Abduljabbar A. H. , and Jarzem P. , Pulmonary Artery Cement Embolism After a Vertebroplasty, Case Reports in Orthopedics. (2015) 2015, no. 1, 10.1155/2015/582769, 582769. 26221556 PMC4499392

[35] Chai T. and Shroff G. S. , Cement Pulmonary Embolism After Percutaneous Vertebral Augmentation in a Patient With Pathologic Lumbar Fracture From Metastatic Breast Cancer, PM and R. (2016) 8, no. 5, 488–490, 10.1016/j.pmrj.2015.12.003. 26724427

[36] Gabe L. M. and Oliva I. B. , Pulmonary Cement Embolism, The American Journal of Medicine. (2016) 129, no. 11, e279–e280, 10.1016/j.amjmed.2016.05.025. 27296328

[37] Gorospe L. , Blanchard-Rodríguez M. J. , and Chinea-Rodríguez A. , Cement Pulmonary Embolism After Percutaneous Vertebroplasty in Multiple Myeloma, Asian Cardiovascular and Thoracic Annals. (2016) 24, no. 4, 400–401, 10.1177/0218492314558521. 25378662

[38] Memarpour R. , Tashtoush B. , Nasim F. , Grobman D. , Upadhyay B. K. , and Rahaghi F. , A Deep-Sea Diver With Cement Pulmonary Embolism, Undersea & Hyperbaric Medicine. (2016) 43, no. 3, 249–55. 27416693

[39] Chang C.-Y. and Huang S.-F. , Asymptomatic Pulmonary Cement Embolism, Canadian Medical Association Journal. (2017) 189, no. 14, E543–E543, 10.1503/cmaj.160579. 28396332 PMC5386849

[40] Mansour A. , Abdel-Razeq N. , and Abuali H. , et al.Cement Pulmonary Embolism as a Complication of Percutaneous Vertebroplasty in Cancer Patients, Cancer Imaging. (2018) 18, no. 1, 1–6, 10.1186/s40644-018-0138-8. 29422089 PMC5806228

[41] Kong M. , Xu X. , Shen J. , Liu Q. , and Wang G. , Clinical Characteristics and Management of Cardiac and/or Pulmonary Cement Embolus After Percutaneous Vertebroplasty: A Single Center Experience, Annals of Translational Medicine. (2019) 7, no. 16, 10.21037/atm.2019.06.81. PMC6736822 31555686

[42] Ross J. , Bhatia R. , Hyde T. , and Dixon G. , Pulmonary Embolism With Coexistent Incidental Pulmonary Cement Embolism Post Vertebroplasty, BMJ Case Reports. (2021) 14, no. 3, 10.1136/bcr-2020-237449. PMC7934751 33664025

[43] Lubinus Badillo F. , Bula Álvarez M. A. , Sáenz Sandoval E. , and Barragán Sandoval J. C. , Cement Pulmonary Embolism After Vertebroplasty: Case Report and Literature Review, Revista Colombiana de Radiología. (2022) 33, no. 4, 5875–5877, 10.53903/01212095.210.

[44] Lenga P. , Bajwa A. A. , and Schneider T. , et al.High Rate of Pulmonary Cement Embolism After Cement-Augmented Pedicle Screw Fixation: A 12-Year Single-Center Study, Journal of Neurological Surgery Part A: Central European Neurosurgery. (2024) 85, no. 2, 117–125, 10.1055/s-0043-1761943. 36828012

[45] Fish A. M. , Kirupaharan P. , and Scharf M. L. , An Incidental Finding of Pulmonary Cement Embolism 4 Weeks After Vertebroplasty in a 50-Year-Old Man With Multiple Myeloma, American Journal of Case Reports. (2023) 24, 10.12659/AJCR.941716. PMC10704105 38037306

[46] Beup M. B. and Gregorio R. , Cement Pulmonary Embolism as a Complication of Vertebroplasty in a Newly Diagnosed Non-Small-Cell Lung Cancer, Chest. (2023) 164, no. 4, A6047–A6048, 10.1016/j.chest.2023.07.3898.

[47] Ates F. , Kilincer A. , Cebeci H. , and Durmaz M. S. , Radiologic Features of Pulmonary Cement Embolism After Percutaneous Vertebroplasty in a Case and Review of Literature, Anatolian Current Medical Journal. (2021) 3, no. 1, 81–84, 10.38053/acmj.815944.

[48] Krueger A. , Bliemel C. , Zettl R. , and Ruchholtz S. , Management of Pulmonary Cement Embolism After Percutaneous Vertebroplasty and Kyphoplasty: A Systematic Review of the Literature, European Spine Journal. (2009) 18, no. 9, 1257–1265, 10.1007/s00586-009-1073-y. 19575243 PMC2899525

[49] Malik M. K. , Wroblewski I. , and Darki A. , Pulmonary Cement Embolism After Vertebroplasty, Cureus. (2023) 15, no. 5, 10.7759/cureus.39194. PMC10276658 37332403

[50] Rahimi B. , Boroofeh B. , Dinparastisaleh R. , and Nazifi H. , Cement Pulmonary Embolism After Percutaneous Vertebroplasty in a Patient With Cushing’s Syndrome: A Case Report, Respiratory Medicine Case Reports. (2018) 25, 78–85, 10.1016/j.rmcr.2018.06.009. 30073141 PMC6068333

[51] Zidan I. , Fayed A. A. , and Elwany A. , Multilevel Percutaneous Vertebroplasty (More Than Three Levels) in the Management of Osteoporotic Fractures, Journal of Korean Neurosurgical Society. (2018) 61, no. 6, 700–706, 10.3340/jkns.2017.0253. 29940724 PMC6280054

[52] Padovani B. , Kasriel O. , Brunner P. , and Peretti-Viton P. , Pulmonary Embolism Caused by Acrylic Cement: A Rare Complication of Percutaneous Vertebroplasty, American Journal of Neuroradiology. (1999) 20, no. 3, 375–377. 10219399 PMC7056072

[53] Sun H.-B. , Jing X.-S. , Shan J.-L. , Bao L. , Wang D.-C. , and Tang H. , Risk Factors for Pulmonary Cement Embolism Associated With Percutaneous Vertebral Augmentation: A Systematic Review and Meta-Analysis, International Journal of Surgery. (2022) 101, 10.1016/j.ijsu.2022.106632, 106632. 35452848

[54] Hsieh M.-K. , Kao F.-C. , and Chiu P.-Y. , et al.Risk Factors of Neurological Deficit and Pulmonary Cement Embolism After Percutaneous Vertebroplasty, Journal of Orthopaedic Surgery and Research. (2019) 14, no. 1, 1–8, 10.1186/s13018-019-1459-4. 31783861 PMC6884871

